# Sperm Lipid Markers of Male Fertility in Mammals

**DOI:** 10.3390/ijms22168767

**Published:** 2021-08-16

**Authors:** Shuwen Shan, Fangzheng Xu, Marc Hirschfeld, Bertram Brenig

**Affiliations:** Institute of Veterinary Medicine, Faculty of Agricultural Sciences, Georg-August-University of Goettingen, 37077 Göttingen, Germany; shuwen.shan@stud.uni-goettingen.de (S.S.); fangzheng.xu@stud.uni-goettingen.de (F.X.); marc.hirschfeld@uni-goettingen.de (M.H.)

**Keywords:** sperm membrane, lipid markers, male fertility, mammals

## Abstract

Sperm plasma membrane lipids are essential for the function and integrity of mammalian spermatozoa. Various lipid types are involved in each key step within the fertilization process in their own yet coordinated way. The balance between lipid metabolism is tightly regulated to ensure physiological cellular processes, especially referring to crucial steps such as sperm motility, capacitation, acrosome reaction or fusion. At the same time, it has been shown that male reproductive function depends on the homeostasis of sperm lipids. Here, we review the effects of phospholipid, neutral lipid and glycolipid homeostasis on sperm fertilization function and male fertility in mammals.

## 1. Introduction

Spermatozoa are characterized as specified haploid cell types that lack most organelles and DNA transcription, resulting in the arrest of protein synthesis and vesicle transport [1]. Spermatozoa possess glycolytic and respiratory capacities, which are predominantly related to the need to maintain cell motility [2]. Additionally, as research progressed, spermatozoa were found to feature lipid synthesis, such as de novo synthesis of phosphatidylcholine [2,3]. The organization of eukaryotic cells depends to a large extent on the structure and function of membranes, based upon the intrinsic properties of membrane lipid components [4]. Spermatozoa lack typical membrane-structured organelles such as the endoplasmic reticulum and Golgi apparatus. The main membrane structures are used to separate cellular regions, such as plasma membrane, outer and inner acrosomal membrane and nuclear envelope (reviewed in [1]). The lipid distribution of spermatozoa can be described with a number of features, including (1) the lipid compositions of the sperm head and tail are different [5]; (2) the plasma membrane of the spermatozoa head shows lateral heterogeneity of surface molecular topology [6,7]; and (3) lipids are also characterized by asymmetric distribution in the membrane bilayer structure [1].

Alterations of lipid components in membranes are usually related to their physiological requirements. For example, the release, modification and adsorption of lipids occur during the transfer of sperm cells through the epididymis [1,8]. Extracellular vesicles (EV), a type of lipid vesicle, are present in the epididymis and seminal fluid. They transport some of the proteins and small RNA secreted by the epididymis or prostate to the sperm, which is critical for fertilization [9,10]. The lipid composition of EV differs in different epididymal regions [11,12]. When sperm enter the female reproductive tract, the lipid composition changes again. In vitro studies revealed that cervical mucus may select different sperm subpopulations based on lipid levels or directly affect the lipid composition of sperm when migrating through the female reproductive tract [13]. Phosphatidylcholine (PC) levels increased when sperm were exposed to female reproductive tract secretions in vivo [14]. As the sperm pass through the utero-tubal junction, they attach and aggregate along the epithelium of the isthmus until ovulation. Spermatozoa are then released from the epithelium and continue to migrate towards the oocyte [15]. Lipids are involved in the interaction of sperm with the oviduct, e.g., anandamide inhibits sperm binding and induces the release of sperm from the oviductal epithelium [16]. At the appropriate time and place sperm cells undergo capacitation and trigger the acrosome reaction (AR), eventually penetrating the zona pellucida (ZP) to fuse with the egg cell [15,17]. During these processes, the plasma membrane lipids undergo another series of alterations [18,19,20]. These, for instance, include the disruption of lipid composition and transmembrane phospholipid asymmetry, lateral diffusion of phospholipids, loss of cholesterol and reorganization of detergent-resistant structural domains observed during the capacitation process [18,21,22]. Subsequent to AR and ZP penetration, the equatorial region of the sperm cell head plasma membrane is involved in the fusion process with the oolemma, the oocyte plasma membrane (reviewed in [1]).

The functions of lipids in male fertility have received increasing attention, with many excellent studies focusing on this subject. In this review, we highlight the important role of lipid classes in sperm during fertilization. In addition, the relationship between plasma membrane lipids and male fertility is discussed, and potential lipid markers of male subfertility and infertility are emphasized. Lipids in sperm are primarily discussed, but minor contents of lipids in seminal plasma and testis are also included.

## 2. Lipid Composition of the Sperm Cell

The lipid composition of the sperm plasma membrane has been elucidated for several mammalian species such as sheep [23,24], cattle [25,26,27], pig [2,14,28], mouse [29,30], rabbit [31], rat [32], horse [33], human [28], ruminantia (cattle, roe deer and Klipspringer) and feloideae species (domestic cat, Siberian tiger and fosa) [34]. Although there is considerable diversity among different mammalian species, in general, the plasma membrane contains about 70% phospholipids, 25% neutral lipids and 5% glycolipids (on molar base) [35]. In contrast to other cell types, the lipid composition of spermatozoa shows some specific characteristics. Spermatozoa have a higher proportion of neutral lipids, especially large amounts of diacylglycerol (DAG) [26,33]. Phospholipids are characterized by a large proportion of alkenyl phospholipids (plasmalogens) in choline- and ethanolamine-containing glycerol phosphates, and high proportions of highly unsaturated fatty acids such as arachidonic (20:4), docosapentaenoic (22:5) and docosahexaenoic acid (22:6) [2,36,37]. Recently, the lipid component (O-acyl)-u-hydroxy-fatty acids (OAHFA) has been identified for the first time in spermatozoa, with a carbon chain length up to 52. It was localized to the head of sperm instead of the tail region [33].

### 2.1. Phospholipids

**Figure 1:**
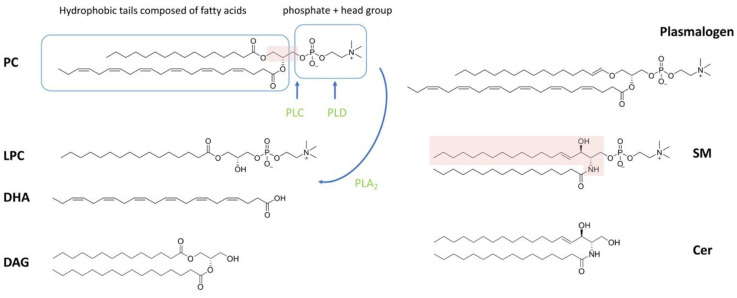
Chemical structure of lipids. The pink shading in phosphatidylcholine (PC) shows the glycerol backbone. The pink shading in sphingomyelin (SM) shows the sphingoid backbone. The blue line boxes show the main parts of PC, the hydrophobic tail and the polar head, respectively. The distinction of PC and plasmalogen is whether the linkage at sn-1 is through an ether bond or ester bond. PC can be hydrolyzed by phospholipase A_2_ (PLA_2_) to LPC and fatty acid. Phospholipase C (PLC) and phospholipase D (PLD) act on the phosphate group and headgroup of phospholipids to break down phospholipids into DAG and PA, respectively. Phospholipases are shown in green and blue straight arrows indicate positions of catalysis.

Of the lipid types present in spermatozoa, phospholipids account for the largest proportion. Phospholipids can be classified as phosphoglycerolipid (also known as glycerophospholipids (GPLs)) and sphingomyelin (SM), depending on whether the backbone is glycerol or sphingosine (Figure 1). Phosphoglycerolipid can be further categorized into different subclasses depending on the different molecules attached to the glycerol backbone. The sn-1 or sn-2 position of the glycerol backbone is connected to the aliphatic acyl-, alkyl- or alkane/alkenyl- chain; the sn-3 position is combined with phosphoric acid and its derivatives [1,38]. The common types of phosphoglycerolipids in most mammals are phosphatidylcholine, phosphatidylethanolamine (PE), phosphatidylinositol (PI) and phosphatidylserine (PS), depending on the attached polar molecule [14,33,39]. Fatty acids (FAs) esterified on phospholipids can be divided into two categories based on the presence or absence of double bonds in the chain structure: (1) saturated fatty acids without double bonds (SFAs), or (2) monounsaturated fatty acids (MUFAs) with a single double bond and polyunsaturated fatty acids (PUFAs) with two or more double bonds [40].

Ether phospholipids are a subclass of GPLs with alkyl or alkenyl chains attached to the glycerol backbone at sn-1 by ether bonds, rather than by ester bonds (Figure 1). The sn-2 position of ether phospholipids generally has an ester-linked acyl chain, same as in diacyl phospholipids [41,42]. Ether phospholipids classify as plasmanyl (1-alkyl-2-acyl) and plasmenyl (1-alkenyl-2-acyl), the latter also being known as plasmalogen [42]. It is noteworthy that plasmalogens represent a high percentage of choline- and ethanolamine-containing phosphoglycerides in sperm, accounting for 50% of the total mass in some species such as human, pig, rat, ruminantia and feloideae [5,28,34].

The GPL-derived signaling molecules consist of lysophosphatidylcholine (LPC), phosphatidic acid (PA), lysophosphatidic acid (LPA) and diacylglycerol (DAG) [43]. Lysophospholipids (LPL) are basically obtained by the selective loss of a fatty acyl residue from phospholipids induced by enzymes and/or reactive oxygen species (ROS). The main enzyme that induces LPL formation is phospholipase A2 (PLA2). In addition, phospholipase C (PLC) and phospholipase D (PLD) catabolize phospholipids into DAG and PA, respectively (reviewed in [44]) (Figure 1). The SM-derived compounds are sphingosylphosphorylcholine (SPC), ceramide-1-phosphate (C1P), sphingosine (Sph), sphingosine-1-phosphate (S1P) and ceramide (Cer), which also are important signaling molecules [38,43]. LPC, LPA, SPC, Sph and S1P readily leave the membrane and signal through the relevant membrane receptors as they carry only one fatty acid chain [45]. In contrast, PA, DAG, C1P and Cer stay in the membrane and recruit cytosolic factors with signaling functions [46].

### 2.2. Neutral Lipids

Cholesterol and diacylglycerol are the main neutral lipids in the sperm plasma membrane. Neutral lipid composition varies between species, individual males and between ejaculates [35]. Cholesterol concentrations in the seminal plasma of many species, such as rams, boars, stallions, human and domestic cats, have been investigated [37,47,48,49]. The synthesis and metabolism pathways of DAG in cells are summarized in [50].

### 2.3. Glycolipids

Glycolipids are an important class of lipids present in sperm membranes, accounting for about 5-8% of total polar lipids in mammalian species [37]. Glycosphingolipids are formed by the addition of glycosidic head groups to ceramides. A more specific one is the glycolipid seminolipid, which contains sulfogalactosylglycerol in its molecular structure. Sulfogalactosylglycerolipid (SGG), also known as seminolipid, is the major anionic glycolipid found exclusively in the plasma membrane of mammalian spermatozoa [51].

## 3. The Role of Lipids in Fertilization and Their Indicative Function in Reduced or Defective Male Fertility

The composition and structure of the sperm plasma membrane are essential in the process of spermatogenesis and sperm maturation [52,53]. During sperm maturation in the epididymis, cholesterol sulfate may act as a membrane stabilizer and enzyme inhibitor [54]. In semen, the stability of sperm membranes relies on the binding of choline phospholipids to seminal plasma proteins, which prevent the movement of phospholipids [55,56,57]. During the migration through the female reproductive tract, sperm undergo a series of biochemical and ultrastructural changes, including changes in the lipid composition of the plasma membrane (reviewed in [58]). For instance, loss of asymmetric transbilayer distribution and the substantial loss of cholesterol and phospholipid occur during capacitation and acrosome reaction (AR) [59,60,61]. The effluxed cholesterol can be bound by albumin and high-density lipoprotein in the uterine and follicular fluid [59,62]. The decrease in phospholipids is mainly due to the breakage of their hydrophobic tails and thus degradation to lysophospholipids and free FAs (22:4 n-9, 22:5 n-6) [19]. Lysophospholipids as signaling phospholipids affect male fertility [38]. Free fatty acids can increase sperm motility, viability and promote AR [63]. During capacitation and AR, sphingosine is hydrolyzed to ceramides, with the main changes occurring in species with very-long-chain polyolefin fatty acids [19]. Loss of cholesterol and phospholipids during capacitation is thought to be associated with phosphorylation of proteins occurring in the tail region of sperm cells, while lipid metabolites produced in AR accumulate in the sperm head and are thought to be involved in later fertilization processes [19,64]. The lipid markers associated with sperm function and fertility are summarized in Table 1.

### 3.1. Phospholipids

#### 3.1.1. Diacyl Phospholipids

PC is the major class of GPL found in all mammalian cells and plays key roles in membrane structure, cell signaling and cell death (reviewed in [50]). In sperm, PC was reported to be associated with sperm motility [65], and to be involved in the acrosome reaction [76]. PS and PE are metabolically relevant membrane aminophospholipids. Their metabolism and function in mammalian cells were well summarized in [77]. PE in sperm membranes can bind to lipocalin 2 in the female reproductive tract, and lipocalin 2 can induce lipid raft reorganization and cholesterol efflux [78]. PS is essential for sperm-egg fusion [79]. PS exposure is also associated with plasma membrane integrity and sperm apoptosis [80,81]. PI and its derivatives are important signaling factors in cells [82,83].

Abnormal levels of PC and PE can be used as markers of the sperm fertilizing ability. Some specific lipid molecules have been suggested to be markers of sperm motility, such as PC 38:4 (composed of stearic and arachidonic fatty acids), PC 36:1 (stearic and oleic fatty acids), PE 34:4 (myristic and arachidonic fatty acids), glycerophosphatidic acid 36:4 (palmitic and arachidonic fatty acids), which showed a high frequency in the plasma membrane of motile sperm [65]. Compared with the control group, the PC content of seminal plasma in obstructive azoospermia human patients and vasectomy patients was significantly lower [66,67]. In patients with idiopathic infertility, decreased sperm PC content has also been reported [68]. There was a significant difference in the seminal plasma PE:PC ratio between patients with spermatogenic failure and obstructive azoospermia [66]. In addition, the ratio of PC to PE is considered to be a key regulator of cell membrane integrity, with a decrease in this ratio leading to loss of membrane integrity [84]. PC is often used as a “protective agent”, for example to prevent ultrastructural damage of sperm caused by cold shock [85,86]. Compared to fresh semen, the plasma membrane of frozen spermatozoa showed a significant decrease in phospholipid, PS, PE and phosphatidylglycerol content, while the content of diphosphatidylglycerol and the ratio of cholesterol to phospholipid increased significantly [69]. In addition, PC is involved in events associated with progesterone-induced rise in intracellular free Ca^2+^ and enhances acrosome responsiveness [76].

**Figure 2:**
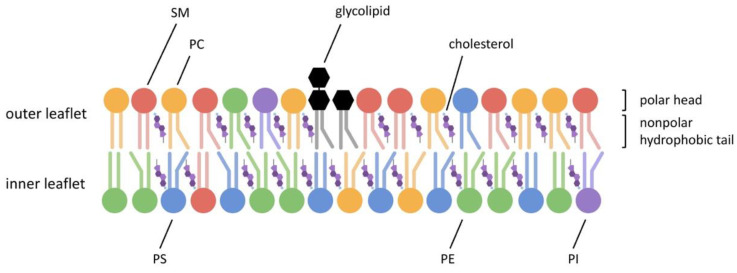
Phospholipid asymmetry of sperm plasma membrane. Sphingomyelin (SM) and phosphatidylcholine (PC) are mainly present in the outer leaflet, while phosphatidylethanolamine (PE) and especially phosphatidylserine (PS) are located in the inner leaflet. Glycolipids are usually found in the outer leaflets of plasma membranes and are mainly used to maintain membrane stability and facilitate intercellular communication. Cholesterol molecules are randomly distributed over the phospholipid bilayer, binding the phospholipids together and limiting the fluidity of the membrane. During capacitation, the sperm plasma membrane first undergoes a substantial loss of cholesterol, accompanied by a decrease in phospholipids and loss of asymmetric distribution of phospholipids.

Phospholipid asymmetry, in which the lipid composition of the plasma membrane differs between the two leaflets (Figure 2), is a common feature of cell membranes. It has also been reported in several mammalian spermatozoa (ram [87,88], bull [89,90], goat [91], boar [92]).

In spermatozoa with intact membranes, SM and PC are mainly present in the outer leaflet while PE and especially PS are located in the inner leaflet [92]. The reduction of phospholipid asymmetry is thought to be related to sperm capacitation and sperm-egg fusion in boars [93]. Aminophospholipid transporter (flippase) located in the acrosomal region can maintain phospholipid asymmetry, and its activity depends upon bicarbonate ions (reviewed in [94]). In previous studies in humans and boars, PS exposure from plasma membrane was often considered a marker of dead or non-viable sperm [81,95,96,97]. When PS is transferred from the inner to the outer leaflet because of disturbed membrane integrity, it can be detected by annexin V. Annexin V is a PS-affinity protein that cannot pass through the intact plasma membrane [98]. The integrity of sperm membrane is critical to sperm fertility as it has been reported to be associated with sperm motility, capacitation and penetration of oocytes [99]. For example, PS-negative sperm have been reported to have higher fertilizing potential in hamster [100] and human [101]. However, PS has also been demonstrated to be transiently exposed to live cells under certain conditions [102,103,104]. Rival et al. [79] have illustrated that PS on sperm and PS receptors (BAI1, CD36, Tim-4, and Mer-TK) on eggs are essential for fertilization.

Currently, most studies of PI on sperm function have focused on phosphoinositides (also known as phosphatidylinositol phosphates (PIPs)), the phosphorylated forms of PI, and the corresponding kinases and phosphatases. Modification of positions 3, 4 and 5 of the inositol ring of PI can generate and interconvert into seven PIP types [105]. The critical role of PIPs in the maintenance of germinal stem cells, proliferation and survival of spermatogonia, spermatogenesis and maturation, and production of motile spermatozoa has been summarized in [105]. Different involved enzymes in the PIP pathway also have an effect on fertility. For example, phosphatidylinositol 3-kinases (PI3Ks), which phosphorylate the 3-position hydroxyl group of the inositol ring of PI, have a negative role in the development and maintenance of human sperm motility [106]. Similar results were obtained in boar spermatozoa, where PI3-K may regulate the negative effects on sperm motility by inhibiting the cAMP/PKA activation pathway [107]. However, a certain level of PI3K expression is necessary to maintain sperm fertilization ability. PI3K can be used as an additional molecular marker for the diagnosis of male infertility [108]. PI3K protects sperm from spontaneous AR, which can reduce fertilization rates [109]. In addition, phosphatidylinositols, as membrane recognition sites, play a key role in membrane fusion [110,111]. Similarly, PIs are involved in the sperm-egg fusion. PIP2 is involved in the pathway of intracellular Ca^2+^ oscillations and egg activation triggered by sperm-specific phospholipase C-ζ [112].

A recent study in rat sperm detected a fully saturated species of cardiolipin (CL), tetrapalmitoyl-CL (TPCL), which was assembled in lipid rafts in the acrosome [113]. Cardiolipin has long been considered a mitochondria-specific phospholipid that is abundant in the sperm tail. The presence of TPCL in the acrosome implies that mitochondria-derived membranes may be involved in the construction of the acrosome [113].

#### 3.1.2. Ether Lipids

It is also known that animal spermatozoa contain very large amounts of plasmalogens [114], which have been reported to accumulate in the sperm head [5]. During sperm maturation, the distribution of plasmalogen fatty acid chains changed significantly, with a decrease in C18:1 and C20:4 and an increase in C22:5 and C22:6 [32,115,116]. Plasmenyl and plasmanyl linked GPLs have been reported to play important roles during the membrane fusion, in membrane fluidity, in regulation of protein activity, and protection of membranes from oxidative damage [117,118,119,120,121]. The functions of plasmalogens in sperm are thought to be maintenance of motility [65] and antioxidation [122]. Ether lipids have been considered to be involved in the formation of macro- and microdomains, which are required for the compartmentalization of highly polarized sperm membrane [123]. In addition, since ether linkages are not readily cleaved by lipase action, ether lipids may maintain stability of sperm membrane [124,125].

Recent studies in human have demonstrated the antioxidant function of plasmalogens in sperm. The first protective characteristic is that plasmalogen contains a vinyl-ether moiety at the sn-1 position that is sensitive to oxidation, thus conferring potentially strong antioxidant properties to plasmalogen [126,127,128]. The interaction of singlet oxygen with the plasmalogen is significantly faster than that of other lipids. Plasmalogens protect unsaturated membrane lipids from oxidation by singlet oxygen when the oxidation products are not excessively cytotoxic [128]. Hypochlorous acid attacks the vinyl-ether bond of plasmalogen can produce glycerophosphocholine and glycerophosphoethanolamine [127]. The second protective feature is that plasmalogens usually bound to high levels of highly unsaturated acyl chains, especially docosahexaenoyl (22:6) and docosapentaenoyl residues (22:5) [41,71]. Plasmanyl-PC 40:4 and plasmenyl-PC 40:5 have been detected to be lipid markers of sperm motility [65]. In fact, reduced sperm motility is possibly a sensitive indicator of lipid peroxidation [129,130,131,132,133,134]. Therefore, the detection of plasmalogens in motile sperm may also be related to their antioxidant function. The quality of cryopreservation is essential for sperm storage. Plasmalogens can be used as one of the indicators of freezing quality, such as the percentage of C16 plasmalogen/total phospholipids and the percentage of plasmalogens to total phospholipids, which are good indicators of bad freezers [135].

#### 3.1.3. Lysophospholipids

Many studies have demonstrated the physiological functions of lysophospholipids (LPLs) in reproduction, from gametogenesis to embryonic development, as well as their pathological roles [38,136]. More specifically, LPCs have been reported to participate in the acrosomal exocytosis and may be associated with perturbation of the cell membrane during fusion [64,137,138]. Destabilization of the acrosome can induce the release of secretory PLA2 from human spermatozoa and the subsequent production of LPC around the spermatozoa [139]. LPC, LPE and LPI have been reported to promote AR in sperm, but LPS does not, and even inhibits LPC-, LPE- and LPI- mediated AR [61,137]. Lysophospholipids are lipids with positive spontaneous curvature and can inhibit the fusion of protein-free bilayers [140]. Lysophosphatidic acid is a small signaling phospholipid whose receptor-mediated signaling has been shown to affect male fertility [141,142]. Lysophosphatidic acid signaling is involved in germ cell apoptosis and proliferation [143]. In addition, LPA can induce the AR and activation of sperm protein kinase C [144]. Platelet-activating factor (PAF), also known as acetyl-glyceryl-ether-phosphorylcholine, is a signalling phospholipid. PAF has been found to play an important role in reproduction [145]. For example, PAF increases sperm motility and promotes sperm capacitation and acrosome reaction in both human and stallions [146,147].

A certain level of LPL is necessary for physiological sperm function. However, LPL production and reacylation need to be available in a tightly controlled equilibrium to avoid premature sperm instability. Under pathological conditions, deviations from normal levels may be observed, and an increase in sperm LPL could be a signal of reduced fertilization potential. For example, deteriorated membranes of spermatozoa are associated with increased LPC content. The ratio of LPC 16:0 to PC 16:0/22:6 and the ratio of LPC 22:6 to PC 16:0/22:6 were found to be enhanced in sperm cells with a damaged membrane structure compared to normal sperm [70]. Since production of LPL can be induced by ROS, LPL can be used as a marker of sperm oxidation under peroxidative conditions. It has been detected that LPC 22:6 and formyl-LPC 22:6 are highly correlated with oxidative damage caused by sperm storage [71]. Investigation of obese people revealed a significant increase in LPC and SM content of sperm [148], while body mass index showed a significant negative correlation with sperm quality [149,150].

#### 3.1.4. Sphingomyelin and Derivatives

Sphingomyelin and ceramide have been reported to serve many functions of membranes, such as formation of microdomains, membrane vesiculation and fusion, vesicle efflux and vesicle trafficking [151,152,153,154]. The different SM/Cer mass ratios in the model system have a great impact on the membrane properties, such as detergent resistance and mechanical properties [155,156,157]. The localization, synthesis and function of SM and Cer in sperm cells of different developmental stages in the testis have been summarized [158,159]. In mammalian sperm, SM and Cer contain a high percentage of very-long-chain PUFA (VLCPUFA) [158,159]. Cer is mostly concentrated in the tail part of the sperm. The SM in the tail contains mainly saturated fatty acids, while SM in the head contains almost exclusively VLCPUFA [5].

SM is heavily hydrolyzed into Cer during capacitation and the AR process [19]. These metabolites are also critical for fertilization. Cer can induce AR in capacitated sperm and potentiates the sperm response to progesterone [160]. Cer acts as an almost immediate precursor of S1P, which has also been reported to trigger acrosomal exocytosis of sperm via a mechanism involving G-protein-coupled receptors [161]. In addition, Cer plays a role in the induction of germ cell death and can be partially inhibited by S1P [162]. A recent study found that S1P can improve DNA repair in murine spermatozoa [163]. Addition of S1P to epididymal preservation media helps maintain sperm viability in cold-transported epididymides [164].

A recent article summarized how sphingolipids control the function of cilia and microvilli in mammalian cells (including the flagella on sperm cells) [165]. The oscillatory motion of the sperm head on the oocyte plasma membrane has been reported to be a critical factor in fusion initiation during the adhesion phase, in which this oscillatory motion is generated by a specific pattern of flagellar beating [166]. Level of SM containing VLCPUFA (≥28 carbons; 4–6 double bonds) revealed a positive correlation with sperm count and total motile sperm cell number [167].

### 3.2. Neutral Lipids

#### 3.2.1. Cholesterol

The earliest study that revealed the role of cholesterol in sperm capacitation dates back to 1978 [168]. After more than 40 years of research, a large number of studies on the relationship between cholesterol and fertilization have been completed and a good series of review articles have been published. For example, the role of sterols in spermatogenesis and maturation [169]; cholesterol function in sperm capacitation [170]; the redistribution and depletion of cholesterol across the sperm membrane as a key part of sperm preparation for fertilization [171]; and the effect of cholesterol on male fertility [172]. Therefore, the function of cholesterol will not be a main focus of this review.

#### 3.2.2. Diacylglycerols

Goni and Alonso [173] have well summarized the structure and function of DAG in cell membranes. DAGs are a minor component of cell membranes [173], but are highly abundant and diverse in sperm [33], implying an important role for DAGs in sperm function. Diacylglycerols, similar to FA and PE, are negative curvature lipids that promote biological fusion (reviewed in [140]).

The most important function of DAG in sperm is its participation in the acrosome reaction. Diacylglycerols have been reported to be engaged in membrane fusion during acrosomal exocytosis [174,175,176], which is consistent with previous studies in other cell types [177,178,179]. In particular, PC-derived DAG is thought to be related to AR exocytosis in boars [176]. Another study revealed that DAGs promoted intraacrosomal calcium efflux and triggered exocytosis of permeabilized human sperm [180]. Intracellular calcium activates adenylyl cyclase to produce cAMP and activates PLC to hydrolyze phospholipids into inositol trisphosphate (IP3) and DAG. A more detailed mechanism of action is summarized in literature [181,182]. DAGs maintain high levels of IP3 to promote exocytosis of the acrosome. DAGs also can activate Rab3A, thus playing a central role in regulating exocytosis and secretion. DAGs further promote the assembly of the SNARE complex and membrane fusion [180]. Intracellular calcium, cAMP and DAG levels were found significantly increased in cryopreserved sperm [183]. This may explain the higher percentage of capacitated sperm in cryopreserved cells [184]. When taurine or trehalose was added to the freezing medium, the levels of intracellular calcium, cAMP and DAG were significantly reduced and the motility, viability and membrane integrity of spermatozoa were significantly improved after thawing [183]. DAG can be catalyzed by diacylglycerol lipase (DAGL) to 2-arachidonoylglycerol (2-AG), an inhibitor of the sperm calcium channel CatSper, which inhibits sperm activation [185]. In our previous study, a reduction in DAG in sperm was associated with bull idiopathic infertility [72]. Disturbances in the metabolism of lipid including DAG may be a causal factor in infertility.

#### 3.2.3. Fatty Acids

FAs are extensively involved in sperm development, maturation and fertilization events [40]. Many studies have examined the relationship between dietary fatty acids and fertility [186,187]. In immature germ cells, the percentage of saturated and essential fatty acids was higher, while long-chain PUFA was significantly lower when compared to mature spermatozoa [188]. Lenzi et al. [114] summarized and compared the unsaturated fatty acid content of normal spermatozoa and red blood cells and found that spermatozoa possessed a higher proportion of the most representative PUFA (C22:6 n-3). This suggests that spermatozoa have active fatty acid metabolism and are desaturated during spermatogenesis or during maturation. Changes in lipid distribution may be a prerequisite for the ability of spermatozoa to acquire fertilization in the epididymis. The most common functions of FAs in sperm include control of membrane fluidity and antioxidant functions.

Polyunsaturated fatty acids are known to facilitate membrane fluidity and flexibility, which are prerequisites for normal sperm function [114]. For example, in the outer leaflet of the sperm plasma membrane, the PC contains a large number of highly unsaturated acyl chains. This results in a reduction in the effective chain length of these moieties and increases the overall cross-sectional area of the phospholipid. The unsaturated acyl chains affect the interactions with cholesterol and reduce the rigidity effect of the membrane. The high degree of unsaturation, the weaker interaction with cholesterol, and the larger cross-sectional area are indicative for the high mobility of the outer leaflet of membrane [124].

High PUFA levels cause spermatozoa sensitivity to reactive oxygen species. ROS attack PUFA in the cell membrane, leading to a cascade of chemical reactions called lipid peroxidation. Associations between oxidative stress and male infertility were well described by a series of excellent publications [160,189,190,191,192,193,194,195,196], and therefore will not be discussed in detail in this review.

Docosahexaenoic is a very critical component of PUFA, and the metabolism and functions of DHA-containing GPLs in testes have been summarized by Hishikawa et al. [197]. DHA is a requirement for spermatogenesis and sperm maturation (see Section 3.4). There is a positive correlation between DHA content and sperm fertilization ability [75]. Arachidonic acid and DHA acid have concentration-dependent inhibitory effects on human sperm motility [198].

PUFAs can be used as markers of sperm fertility and pathology. Safarinejad et al. [73] compared the composition of sperm FAs in fertile and infertile men and found higher omega-3 FAs in the fertile group but higher levels and percentages of arachidonic acid in the infertile group. Am-In et al. [199] investigated the lipid profiles of normal and low-motility sperm from boars. Docosahexaenoic acid and n-3 PUFA were found to be positively correlated with sperm viability, survival, normal morphology and normal plasma membrane. In contrast, the ratios of saturated fatty acids and n-6/n-3 PUFA were negatively correlated with normal sperm parameters [199]. The differences in sperm motility can be explained by varying antioxidant capacity resulting from the different n-3 PUFA content in the plasma membrane.

### 3.3. Glycolipids

Sulfogalactosylglycerolipid (SGG) is the major sulfoglycolipid of mammalian male germ cells. Knock-out mouse models revealed important roles of SGG sperm function and male fertility, as summarized by Tanphaichitr et al. [200]. Sperm SGG has been reported to be involved in spermatogenesis [200] and the binding of sperm and zona pellucida (ZP) [201,202]. Redistribution of glycolipids has been detected during the binding to ZP. Sulfogalactolipids are localized in the apical ridge subdomain of freshly ejaculated sperm cells, and migrate to the equatorial subdomains after sperm binding to ZP [203,204]. It has been proposed that sugar residues exposed on the sperm surface during capacitation are important for subsequent binding to ZP [205]. In inactivated spermatozoa, membrane cholesterol induces a tilt in the glycosphingolipid receptor, rendering it unusable. Cholesterol efflux during capacitation causes a change in glycosphingolipids conformation, exposing sugar residues that can be recognized by lectins in the egg zona pellucida [206]. Sulfogalactosylglycerolipid is considered to be an important biomarker for sperm fertilizing ability [75].

### 3.4. Lipid Metabolism-Related Knockout Mice Model

Lipid-metabolizing enzyme-related knockout mice are good models for studying the role of lipids in spermatogenesis and maturation. Knockout models can also be used to study the effects of lipids on fertility. In this section, some reports of metabolic enzyme knockout mice associated with phosphatidylinositol phosphates, ether lipid, sphingolipids, fatty acids and lysophospholipids are summarized. Mice with deletions of lipid-metabolizing enzymes typically exhibit abnormal spermatogenesis and male infertility. Examples for knockout mouse models related to lipid metabolism are listed in Table 2.

Phosphatidylinositol phosphates (e.g., PI4P, PIP2, PIP3) are membrane lipids that play well-defined roles in different stages of sperm development. More specifically, PIPs are involved in the maintenance of germinal stem cells, proliferation and survival of spermatogonia, cytokinesis of spermatocytes, polarization of sperm, formation of sperm tail, nuclear shaping and production of motile spermatozoa [105]. The relevant gene editing mouse models for the PIP pathway are well summarized in [105]. A recent study found that most of the embryos in a mouse model of PI3K catalytic isoform p110β inactivation were lethal, and homozygous mice that survived to adulthood were male infertile [207]. Inositol polyphosphate 4-phosphatase II (INPP4B) is an enzyme in the phosphatidylinositol signaling pathway. Inpp4b^−/−^ male mice have smaller testes and fewer mature sperm cells than wild-type mice [208].

Mouse models with defects in enzymes involved in ether lipid synthesis show abnormal plasmalogen levels, often manifesting as male infertility [123,209]. For example, dihydroxyacetonephosphate acyltransferase (*DAPAT*) knockout mice exhibited severe phenotypic alterations, including cessation of spermatogenesis and infertility, illustrating the important role of plasmalogen in spermatogenesis [123,210,211]. Peroxin 7 (*Pex7*) knockout mice displayed severely depleted plasmalogens, while adult mutant mice were infertile and exhibited testicular atrophy [212]. Male Tysnd1^−/−^ mice are sterile and have incomplete acrosomes of epididymal sperm, which may be caused by altered components of choline and ethanolamine plasmalogens [213].

Sphingomyelin biogenesis-deficient mouse models revealed the role of SM in spermatogenesis. Sphingolipid synthase 1 (SMS1) catalyzes the de novo synthesis of SM and DAG from Cer and PC [214]. Spermatogenesis defects in Sms1^−/−^ mice manifested by shedding of spermatocytes and spermatids, leading to progressive sterility in homozygote mutant males. They exhibited reduced long-chain unsaturated PCs, LPCs and sphingolipids [215]. Similarly, abnormalities in acid sphingomyelinase (ASM), which catabolizes SM, can also affect SM homeostasis. Abnormalities in sperm morphology, motility and capacitation were observed in ASM knockout mice. Incubation of mutant murine spermatozoa with mild detergent resulted in recovery of morphological abnormalities, suggesting that the sperm bending defect is a direct consequence of membrane lipid accumulation [216].

The structure of fatty acids is usually determined by a combination of two enzymes: membrane-bound fatty acid desaturase 1 and 2 (FADS1 and FADS2) and fatty acid elongases (ELOVL2 and ELOVL4) [217]. In male FADS2 knockout mice, acrosome formation fails during the spermiogenesis of round sperm into elongated sperm, and therefore causes infertility. One reason could be the deficiency of highly unsaturated fatty acids. However, this defect can be reversed by docosahexaenoic acid (DHA) in the diet [218,219,220,221]. Male Elovl2^−/−^ mice, as expected, also displayed arrested spermatogenesis and infertility, which supports the idea that PUFAs with 24–30 carbons of the ω-6 family are required for normal sperm formation and male fertility. Although both FADS2 and ELOVL2 are involved in the formation of FAs, male sterility in Elovl2^−/−^ mice could not be restored by diet, in contrast to Fads2^−/−^ mice [222,223].

In addition, lysophosphatidic acid acyltransferases (LPAATs) can use lysophosphatidic acid as an acyl receptor to regulate the FA composition of GPLs. Recent studies have shown that male LPAATs knockout mice are completely sterile, characterized by abnormal spermatogenesis due to defects in eliminating excess cytoplasmic components [224]. The marked reduction in DHA-containing GPLs in LPAATs knockout mice may affect membrane flexibility, which is necessary for endocytosis in the final step of spermatogenesis [197]. DHA also plays roles in the maturation of spermatozoa in the epididymis. During sperm transfer in the epididymis, the acyl groups of PC on the membrane changes from oleic, linoleic and arachidonic acid (AA), to docosapentaenoic (DPA) and DHA [11]. Phospholipase A2 group III (Pla2g3) is expressed in epididymal epithelial cells. Pla2g3^−/−^ mouse sperm exhibited decreased motility and fertility due to impaired acyl remodeling of oleic, linoleic and AA to DPA and DHA in epididymis [225]. Delta-6 desaturase-null mice exhibited an arrest at the late stage of spermatogenesis and infertility due to defective synthesis of AA, DHA and n6-DPA [74].

**Table 2:** Effects of lipid metabolism-related knockout in mice on reproduction.

Lipid Metabolism Knockout Mouse Model	Lipid Alterations	Effect on Fertility	Reference
p110β inactivation	-	Male infertility	[207]
Inpp4b^−/−^	-	Impaired male germ cell differentiation	[208]
DAPAT^−/^^−^	Absence of ether lipid	Spermatogenesis is arrested during the late pachyteneand of the spermatid stage; male infertility	[123,210,211]
Pex7^−/^^−^	Depleted plasmalogen levels	Testicular atrophy; male infertility	[212]
Tysnd1^−/^^−^	Altered components of choline and ethanolamine plasmalogens	Incomplete acrosomes of epididymal sperm; male sterile	[213]
Sms1^−/^^−^	Reduction in long-chain unsaturated PCs, LPCs and sphingolipids	Shedding of spermatocytes and spermatids during spermatogenesis; progressive male sterility	[215]
ASM^−/^^−^	Increased levels of sphingomyelin and cholesterol	Abnormalities of sperm morphology, motility and capacitation	[216]
FADS2^−/^^−^	Deficiency of highly unsaturated fatty acids	Failure of acrosome formation during the spermiogenesis of round sperm into elongated sperm; male infertility	[218,219,220,221]
Elovl2^−/^^−^	Reduction in PUFAs with 24–30 carbons of the ω-6 family	Arrested spermatogenesis; male infertility	[222,223]
LPAATs^−/^^−^	Reduction in DHA-containing GPLs	Abnormal spermatogenesis due to defects in eliminating excess cytoplasmic components; male sterility	[224]
Pla2g3^−/^^−^	Impairment of acyl remodeling of oleic, linoleic and arachidonic acids to docosapentaenoic and docosahexaenoic acids	Decreased sperm motility and fertility	[225]
D6D^−/^^−^	Defective synthesis of arachidonic acid (AA), DHA and n6-DPA	Arrest at late stage of spermatogenesis; male infertility	[74]

p110β: PI3K catalytic isoform; INPP4B: inositol polyphosphate 4-phosphatase II; DAPAT: dihydroxyacetonephosphate acyltransferase; Pex7: peroxin 7; Tysnd1: trypsin domain-containing 1; Sms1: sphingomyelin synthase 1; ASM: acid sphingomyelinase; FADS2: fatty acid desaturase 2 (alias names: D6D); Elovl2: elongation of very-long-chain fatty acid 2; LPAAT: lysophosphatidic acid acyltransferase; Pla2g3: phospholipase A2 group III; D6D: delta-6 desaturase.

## 4. Conclusions

Sperm–egg union is one of the most important events in sexually reproducing organisms. Lipids, as major components of the membrane, maintain sperm integrity, control membrane fluidity, provide functional cell membrane microstructural domains and provide signaling molecules. Lipids are extensively involved in a wide range of processes, from spermatogenesis and maturation, to meeting and binding to the egg cell. In this review, we summarized the effects of different lipid types described so far on the events during fertilization. We also list the effects of lipid deficiency on spermatogenesis, maturation and fertilization ability in a lipid metabolism-related knockout mouse model.

Each lipid class does not act in a separate manner; they rather participate together in concerted complex pathways through synthesis, metabolism or interconversion. Sperm morphology, motility and oxidative stress damage were frequently investigated in numerous studies on male fertility. Corresponding highly relevant lipids such as unsaturated fatty acids, plasmalogens and lysophospholipids were studied with high frequency. Unsaturated fatty acids and plasmalogens perform major antioxidant functions, while lysophospholipids are markers of sperm oxidation under peroxidative conditions. Phosphatidylcholine and phosphatidylethanolamine are the most important components of the sperm plasma membrane, and therefore their levels and ratios are the most readily detectable indicators of sperm fertilization ability. Diacylglycerols and sulfogalactosylglycerolipids are of interest because of their abnormally high content in sperm or their specific occurrence in germ cells. Phosphatidylinositols, sphingomyelin and its derivatives and lysophospholipids are frequently involved in fertilization as signaling molecules, which are essential for fertility.

Many challenges remain to be solved in the elucidation of lipid functions in fertilization. Some trace amounts of lipids or lipids that play a transient role during fertilization are difficult to detect accurately. In vitro experiments do not always exactly replicate the state of the membrane in the in vivo setting. The development of novel techniques and methods may provide more clues to lipid-regulated fertilization processes and the potential effects of deregulated lipid levels on male mammalian fertility.

## Figures and Tables

**Table 1:** Lipid markers associated with sperm function and fertility.

Lipid Class and Ratio *	Impact or Relevance on Sperm and Fertility	Species	Reference
PC 38:4 (18:0/20:4), PC 36:1 (18:0/18:1), PE 34:4 (14:0/20:4) and glycerophosphatidic acid 36:4 (16:0/20:4)	Presented high frequency in the plasma membrane of motile sperm	dog	[65]
PC content of seminal plasma	Decreased in obstructive azoospermia patients and vasectomy patients	human	[66,67]
PE:PC ratio of seminal plasma	Significant differences between patients with spermatogenic failure and obstructive azoospermia	human	[66]
PC	Decreased in idiopathic infertility	human	[68]
Plasmanyl-PC 40:4 and plasmenyl-PC 40:5	Presented high frequency in the plasma membrane of motile sperm	dog	[65]
Phospholipid, PS, PE and phosphatidylglycerol	Decreased in the frozen spermatozoa	ovine	[69]
Diphosphatidylglycerol and the ratio of cholesterol to phospholipid	Increased in the frozen spermatozoa	ovine	[69]
Ratio of LPC 16:0 to PC 16:0/22:6 and ratio of LPC 22:6 to PC 16:0/22:6	Increased in sperm with damaged membrane structure	human	[70]
LPC 22:6 and formyl-LPC 22:6	Increased in sperm with oxidative damage caused by sperm storage	cattle and roe deer	[71]
DAG	Decreased in idiopathic infertility	cattle	[72]
Total lipids, cholesterol, phospholipids, PUFA, DHA and n-3 PUFA	Positively correlated with sperm viability, survival, normal morphology and normal plasma membrane	boar	[73]
SFA and ratios of n-6 to n-3 PUFA	Negatively correlated with normal sperm parameters	boar	[73]
AA	Higher in infertile group than control group	boar	[73]
Omega-3 FAs	Lower in infertile group than control group	human	[74]
SGG	Higher in the group of sperm with higher fertilization capacity	mouse	[75]

PC: phosphatidylcholine; PE: phosphatidylethanolamine; PS: phosphatidylserine; LPC: lysophosphatidylcholine; DAG: diacylglycerol; SFA: saturated fatty acid; PUFA: polyunsaturated fatty acid; DHA: docosahexaenoic acid; AA: arachidonic acid; FA: fatty acid; SGG: sulfogalactosylglycerolipid. * Refers to the lipid content of the sperm, except for the additional description.

## Data Availability

Not applicable.
